# Bouncing back or staying inactive? The social inequality in sport participation before, during and after the COVID-pandemic

**DOI:** 10.1080/02614367.2023.2243652

**Published:** 2023-08-26

**Authors:** Remco Hoekman, Malou Grubben, Gerbert Kraaykamp

**Affiliations:** a Department of Sociology, Radboud University Nijmegen, The Netherlands; b Department of Sociology, Mulier Institute, Utrecht, The Netherlands

**Keywords:** sport participation, COVID, educational inequality, financial inequality, dropout

## Abstract

This contribution addresses the development of educational and financial inequality in sport participation in the Netherlands over the course of the COVID-pandemic. More specifically, we deal with the issue whether increased educational and financial inequality during the COVID-pandemic is temporary or becomes permanent after relaxation of the COVID-measures. We expected lower educated and people with financial problems to have less resources to bounce back to sport participation or to continue sport participation in sport over the course of the COVID-pandemic. To test our expectations, we performed multinominal logistic regression analyses on longitudinal data from the Dutch LISS-panel (*n* = 1.157). Our results confirmed that higher educated more often bounced back in their sport participation after COVID. Lower educated and people with financial problems were more likely to completely dropout. Our main conclusion is that educational and financial inequality in sport participation further increased after relaxation of the COVID-measures. This study enhances the understanding of the long-term impact of the COVID-pandemic on inequality in sport participation, and that might lead to more intensified sport promotion policies. Ongoing attention of policymakers for vulnerable groups is required to tackle social inequality in sport participation enlarged by the COVID-pandemic.

## Introduction

In 2020, the World Health Organization (WHO) declared COVID-19 as a global pandemic. The following 2 years were characterised by the implementation of restrictive measures to prevent the spread of the COVID-19 virus, including social distancing, self-quarantining and lockdowns. These preventive measures significantly affected people’s daily lives with levels of loneliness, anxiety, stress and insomnia increasing (Killgore et al., 2020; Rossi et al., 2020; Shevlin et al., 2020).

Sport is often considered relevant to mitigate problems that result from the COVID-pandemic (Lewis et al., 2021; Meyer et al., 2020; Reyes-Molina et al., 2022; Stanton et al., 2020). For these reasons, the Dutch government stressed the importance of sport participation during the COVID-pandemic for all groups in society, with the aim of achieving a more resilient society (RIVM, 2020). In contrast to encouragements to participate in sport, the Dutch government also considered it inevitable to implement restrictions with regard to sport. Dutch COVID-policy measures included a closure of sport clubs and other sport facilities (fully closed, or solely in the evening) and imposed serious limitations on sport together with others.

Several studies in various countries investigated the extent to which restrictive COVID-measures affected individuals’ sport behaviour. A review article of Stockwell et al. (2021) showed that in the majority of these studies a decrease in the level of sport participation and physical activity during the COVID-pandemic is demonstrated. Although the restrictive COVID-measures applied to everyone, research also indicated that social divisions in sport participation intensified during the COVID-pandemic (De Boer et al., 2021; Grubben et al., 2022). Such findings are in line with previous studies on the impact of global crises revealing that vulnerable groups are affected most (Collins & Haudenhuyse, 2015; Roberts, 2015). Only recently, Feder et al. (2023) showed no discernible transformation in the stratification of cultural participation in England during the COVID-pandemic.

Prior studies on sport participation in COVID-times did not reveal whether the impact of the COVID-pandemic on social inequality in sport participation was lasting and stayed even after a relaxation of all COVID restrictions. Yet, it is crucial to determine whether short-term disruptions in sport behaviour caused a long-term expansion of the inequality gap in sports with its adverse effects for individuals and society. In our paper, we aim to fill this lacuna and study to what extent people who dropped out of sports during COVID were able to bounce back to their pre-pandemic sport behaviour after relaxation of the COVID-measures. By using longitudinal information, we determine to what extent individuals in the Netherlands continued their sport participation in the course of the COVID-pandemic and to what extent people who dropped out were resilient to bounce back to pre-pandemic sport participation after relaxation of COVID-measures. In doing so, we establish to what extent increased social divisions in sport participation during the COVID-pandemic are temporary or structural. We here focus on the educational and financial inequality in sport participation.

Overall, our research questions are the following: (1) *To what extent did people drop out over the course of the COVID-pandemic, bounce back after relaxation of the COVID-measures, or continued their sport participation in the course of the COVID-pandemic and* (2) *to what extent do these developments differ by educational level and financial situation?*

To answer our research questions we employed longitudinal data from the Dutch LISS panel that includes information on people’s sport participation before (in 2019), during (in 2021) and after the COVID pandemic (in 2022). People who did not participated in sport in 2019 were not included, since they are not at risk to drop out from sports. In using this unique research design, we aim to contribute to painting a comprehensive picture of the long-term impact of the COVID-pandemic on inequality in sport participation. We further hope this knowledge may be relevant to sport stimulation policy debates.

## Educational and financial inequality in sport participation

After most COVID-restrictions were lifted, including those on participating in sport (in 2022), it was possible to bounce back to pre-pandemic sport behaviours. A general expectation is that especially lower educated and people with financial problems had a relatively hard time doing this. This presumption finds ground in Bourdieu’s concepts of cultural and economic capital (Bourdieu, 1984). Bourdieu (1978, 1984) argued that social differentiation in lifestyle, which includes sport participation, depends on the quantity and type of capital a person possesses (see also Kraaykamp, 2002; Wilson, 2002). The various forms of capital (cultural, economic) closely relate to a person’s habitus that refers to an internalised system of dispositions which engenders all thoughts, perceptions and actions of an individual (Bourdieu, 1984). A person’s habitus is the product of early childhood experiences and various socialisation experiences in a person’s social environment (Reay, 2004). A habitus favouring sports likely make people more resilient to cope with external influences such as COVID and uphold or adapt their preferred sport behaviour.

Here, cultural capital is represented by a person’s educational attainment. Generally, lower educated are believed to have less competency and knowledge about healthy behaviours, as well as on the health promoting effects of sport participation (André et al., 2018; Rademakers, 2014). Moreover, lower educated people are assumed to be less socialised with participating in sport, and therefore participating in sport is less embedded in their internalised system of dispositions (Warde, 2006; Stempel, 2005). We therefore expect that lower educated people feel less urgency to stay active and hold fewer resources to continue sport participation during COVID. Moreover, we expect them to be less inclined to return to sport behaviours after relaxation of the COVID-measures.

Next, a lack of financial resources may also be a barrier to a person’s sport participation (Coalter, 1993; Wilson, 2002). Doing sports requires, for example, the purchase of sport equipment, clothing or a membership fee. During COVID-19 for people with limited financial opportunities, a shift to alternative sport practices and the return to sport after the COVID-pandemic may therefore be less feasible. Additionally, the ending of the COVID-pandemic coincides with an economic and energy crisis which even may exacerbated the role of financial aspects in deciding to sport. We therefore expect people with financial problems to be less resilient to continue their sport participation during the COVID-pandemic and to bounce back less often to pre-pandemic sport participation after COVID.

This leads to the following two hypotheses: (1) *lower educated and people with financial problems have a lower likelihood to continue participation in sport during the COVID-pandemic as compared to higher educated and people without financial problems and* (2) *lower educated and people with financial problems have a lower likelihood of bouncing back to participation in sport after the COVID-pandemic, as compared to higher educated and people without financial problems.*

## Materials and methods

### Data collection

To test our hypotheses, we employ information of two waves (2021 and 2022) from the Dutch Longitudinal Internet Studies for the Social Sciences (LISS). The LISS panel is a nationally representative longitudinal survey of about 5.000 households comprising approximately 7.500 individuals aged 16–69 years old. In our study, we randomly selected one individual per household. To study an individuals’ sport participation over the course of the COVID-pandemic, we merged two LISS waves with elaborate information on sport participation in 2019, 2021 and 2022. In the first wave, collected between 7 June and 27 July 2021, we included retrospective questions on a person’s sport participation in 2019 (before COVID), and in March, April and May 2021 (during COVID). In a second wave, executed between 6 June and 26 June 2022, we approached all respondents who participated in wave 1 with questions on their sport participation in March, April and May 2022 (after COVID). Lower educated men are slightly underrepresented in LISS. To safeguard representativity, we weighted the data for educational level, gender and age group, using population information from Statistics Netherlands. More information about LISS can be found at: www.lissdata.nl.

We only selected respondents in the analyses who indicated to participate in sport before the COVID-pandemic. Our final sample consists of respondents who indicated to participate in sport before COVID, and with valid scores on sport participation questions during and after the COVID-pandemic (*N* = 1.163). We excluded respondents with missing information on educational level, financial situation and relevant controls (0,5% missing). Our final sample consists of 1.157 respondents.

### Measurements

Respondent information on sport participation in 2019, 2021 and 2022 was used to investigate an individuals’ development in sport participation over the course of the COVID-pandemic. To measure sports participation respondents for each year were asked: ‘did you participate in sport?’. The question implies that respondents were allowed to interpret sports as they understood it, making the interpretation of sports subjective. To study an individual’s sport development, we distinguish four possible trajectories: (0) continuous sport participation between 2019 and 2022, (1) dropout in 2021 (people who dropped out during COVID and did not bounce back in 2022), (2) dropout in 2022 (people who participated in sport during COVID, but dropped out afterwards) (3) bounced back in 2022 (people who dropped out during COVID and bounced back). The categorisation of our dependent variable is displayed in Table 1.

**Table 1.** Measurement of sport participation in the course of the COVID-pandemic.

Development sport participation	Before COVID (2019)	During COVID (2021)	After COVID (2022)	%
Continuous sport participation 2019–2022	Active	Active	Active	59.5%
Dropout in 2021	Active	Inactive	Inactive	13.4%
Dropout in 2022	Active	Active	Inactive	11.7%
Bounced back in 2022	Active	Inactive	Active	15.4%

Source: LISS, 2022, *N* = 1.157.

Educational level was measured using standard Dutch qualifications (ISCED levels). Respondents were categorised into three groups based on the highest level of education finished with diploma: (0) lower educated (intermediate secondary education or lower), (1) middle educated (higher secondary education or intermediate vocational education) and (2) higher educated (higher vocational education or university).

Respondents were also asked about their financial situation in March, April and May 2021 and the same period of time in 2022. Six questions referred to whether respondents had a hard time making ends meet, were unable to quickly replace belongings that broke, lend money for necessary expenditures, were behind in paying rent/mortgage or general utilities, had a debt collector at their door and received financial support from friends or family. Possible answers were ‘yes’ and ‘no’. People who scored ‘yes’ at least on one question were coded as having financial problems (1).

We controlled for *gender* (0 = female, 1 = male), *age* and *ethnic background* (Dutch, non-Western and Western). Additionally, we included whether respondents had children living at home (1 = yes). Finally, we took into account whether people suffer from long-term health problems or a handicap (1 = yes). Table 2 displays descriptive statistics.

**Table 2.** Descriptives.

	Minimum	Maximum	Mean	Std. Dev
**Dependent variable**				
Development sport participation				
Continuous sport participation 2019–2022	0	1	.595	.491
Dropout in 2021	0	1	.135	.341
Dropout in 2022	0	1	.117	.321
Bounced back in 2022	0	1	.154	.361
**Independent variables**				
Educational level				
Lower educated	0	1	.140	.347
Middle educated	0	1	.353	.478
Higher educated	0	1	.508	.500
Financial problems during COVID-pandemic (ref.=no)	0	1	.152	.359
Gender (ref.=female)	0	1	.493	.500
Age (mean = 0)	−24.2	29.8	.000	15.969
Ethnic background				
Dutch	0	1	.800	.400
Non-Western	0	1	.111	.315
Western	0	1	.088	.284
Long-term health problems or handicap (ref.=no)	0	1	.216	.412
Children living at home (ref.=no)	0	1	.439	.496

Source: LISS, 2022, *N* = 1.157.

### Analytical strategy

To begin, we present descriptive information on respondent’s development in sport participation over the course of the COVID-pandemic split out for educational level and financial situation. Next, multinomial logistic analyses were conducted to test our hypotheses on inequality in the development of sport participation controlling for all relevant variables. Our dependent variable indicates within-person change in sport participation during the course of the COVID-pandemic. Completely quitting to participate in sport during COVID without bouncing back is taken as a reference category (dropout in 2021). This enables us to investigate educational and financial inequality in sports over the course of the COVID-pandemic.

## Results

### Descriptive results

**Figure 1.**
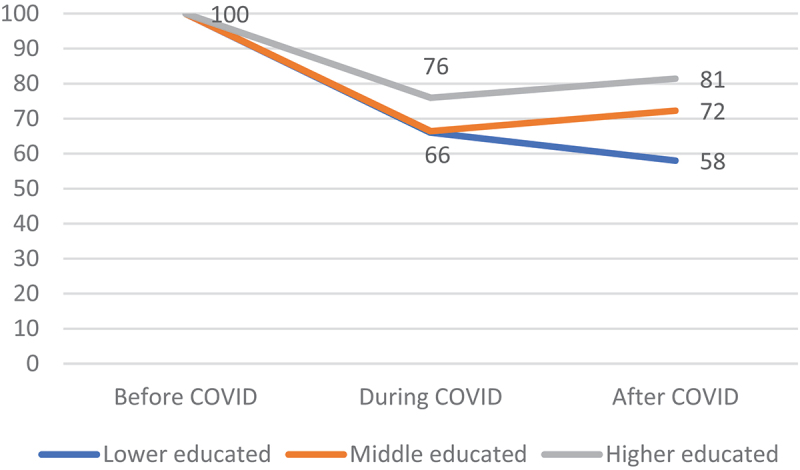
Participation in sport over the course of the COVID-pandemic by educational level (in percentages).

Source: LISS, 2022, *N = 1.157*

**Figure 2.**
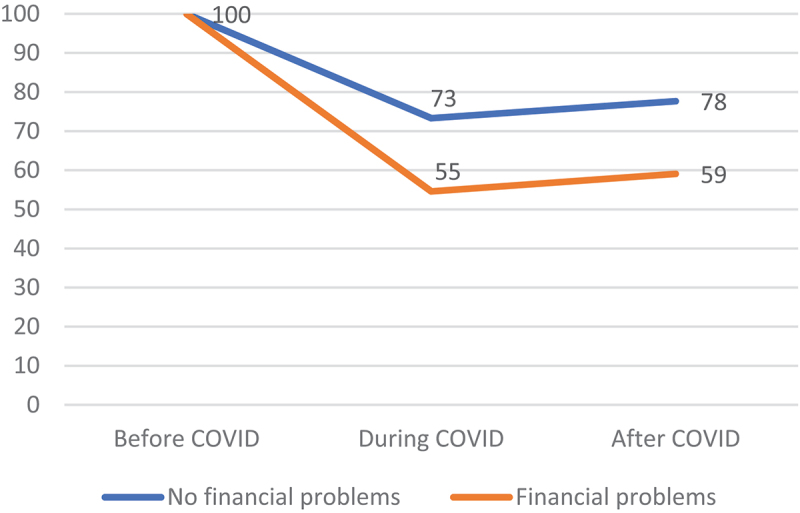
Participation in sport over the course of the COVID-pandemic by financial situation (in percentages).

Source: LISS, 2022, *N = 1.157*

Figure 1 displays that a relatively large proportion of the higher educated (76%) continued to participate in sport during the COVID-pandemic compared to their lower and middle educated counterparts (66%). With regard to the situation after COVID, we especially observed that lower educated did bounce back to a lesser extent than middle and higher educated respondents (after a dropout in 2021). A similar pattern is visible in Figure 2 for an individual’s financial situation. A relatively high percentage of people without financial difficulties (73%) continued to participate in sport during the COVID-pandemic, as compared to people with financial problems (55%). After COVID, it seems that a slightly smaller number of people with financial problems bounced back to sports (4% points for people with financial problems versus 5% points for people without financial difficulties). These results provide a first indication of a rise in educational and financial inequality in sports participation after the COVID-pandemic. Although our descriptive results provide interesting information findings may be affected by confounding aspects. Moreover, descriptive changes do not adequately reflect all developments in an individual’s sport participation between 2019 and 2022 since people can follow various trajectories (Table 1). Our multinomial logistic regression analysis therefore distinguishes all four possible trajectories of sport participation during the course of the COVID-pandemic.

### Multinomial logistic regression analysis

Table 3 reports results of a multinomial logistic regression analysis. We report unstandardised estimates (B’s), and Exp(B’s) which may be interpreted as odds ratios. An Exp(B) greater than 1 indicates a positive effect, while an Exp(B) smaller than 1 refers to a negative effect. A positive effect in a multinomial logistic model may be interpreted as a greater likelihood to being in a certain trajectory of sport participation during the COVID-pandemic, compared to being in the reference category ‘drop out from sports in 2021’, which refers to completely quitting sports and not bouncing back.

**Table 3.** Multinomial logistic regression of development of sport participation in the course of the COVID-pandemic.

	B	s.e.	Exp(B)
**Continuous sport participation 2019–2022**			
Educational level (ref.=lower educated)			
Middle educated	.427	.258	1.532
Higher educated	1.111***	.264	3.039
Financial problems during COVID-pandemic (ref.=no)	−.882***	.236	.414
Gender (ref.=female)	−.058	.186	.943
Age (mean = 0)	.010	.006	1.010
Ethnic background (ref.=Dutch)			
Non-Western	−.766**	.272	.465
Western	.039	.347	1.050
Long-term health problems or handicap (ref.=no)	−.370	.225	.691
Children living at home (ref.=no)	−.155	.199	.856
Intercept	1.265***	.278	
**Dropout in 2022**			
Educational level (ref.=lower educated)			
Middle educated	−.250	.318	.779
Higher educated	.126	.324	1.135
Financial problems during COVID-pandemic (ref.=no)	−.449	.296	.638
Gender (ref.=female)	−.653**	.244	.521
Age (mean = 0)	.001	.008	1.001
Ethnic background (ref.=Dutch)			
Non-Western	.295	.320	1.343
Western	.481	.418	1.618
Long-term health problems or handicap (ref.=no)	.056	.283	1.058
Children living at home (ref.=no)	−.060	.259	.942
Intercept	.227	.342	
**Bounced back in 2022**			
Educational level (ref.=lower educated)			
Middle educated	.862*	.343	2.368
Higher educated	1.283***	.350	3.609
Financial problems during COVID-pandemic (ref.=no)	−.106	.282	.900
Gender (ref.=female)	−.357	.228	.700
Age (mean = 0)	.024**	.008	1.025
Ethnic background (ref.=Dutch)			
Non-Western	−.257	.327	.774
Western	−.031	.419	.969
Long-term health problems or handicap (ref.=no)	−.398	.274	.672
Children living at home (ref.=no)	−.329	.243	.720
Intercept	−.297	.364	
Dropout from sport in 2021 is reference category			

*p<.050, ** p < .010, ***p<.001: *two-tailed test*.

Source: LISS, 2022, *N = 1.157*.

First, we investigate whether higher educated and people without any financial problems are more likely to continue sport participation during the COVID-pandemic compared to completely quitting sport participation during the COVID-pandemic. Our results from Table 3 confirm that, indeed, higher educated are more likely to continue participating in sport versus dropping out in 2021 than lower educated people (Exp(B) = 3.039). The odds ratio of 3.039 indicates that the likelihood of continuous sport participation 2019–2022 versus dropping out in 2021 is more than 3 times larger for higher educated people than for lower educated individuals. Also, people with financial problems are significantly less likely to continue sport participation versus dropping out in 2021 compared to people without financial problems (Exp(B)=.414). This odds ratio of .414 implies that the likelihood of continuous sport participation 2019–2022 versus dropping out in 2021 is more than 2 times smaller for respondents with financial problems compared to the people without financial problems.

Next, we investigate whether the odds of bouncing back in 2022 to sport participation are different for educational groups and people with and without financial problems. Our analysis shows that higher educated indeed are more likely to bounce back to sport participation in 2022 versus dropping out in 2021 compared to lower educated people (Exp(B) = 3.609). This odds ratio indicates that higher educated have a more than three times greater likelihood of bouncing back after the COVID-pandemic than lower educated people. We found no significant differences between people with and without financial problems in their likelihood to bounce back to sports in 2022 versus dropping out completely.

Remarkably, we observe little influence of the control variables we included (Table 3). Only people with a non-Western background (Exp(B)=.465) seem relatively less likely to continue sport participation between 2019 and 2022 versus drop out in 2021, compared to people with a Dutch origin. Also, the likelihood of bouncing back after dropping out during the COVID-pandemic increases somewhat with rising age (Exp(B) = 1.025).

## Discussion and conclusion

In this contribution, we addressed educational and financial inequality in the development of individual’s sport participation in the Netherlands over the course of the COVID-pandemic. Prior research mainly focused on a comparison before and during COVID and established that educational and financial inequality in sport participation increased during the COVID-pandemic (De Boer et al., 2021; Grubben et al., 2022). With our research, we build on these studies by investigating how this inequality has developed after relaxation of the COVID-measures. We consider this to be a meaningful effort, providing information on whether increased educational and financial inequality observed during the COVID-pandemic is temporary because people bounced back or become permanent (people stay inactive) after relaxation of the COVID-restrictions.

In this paper, we answer two research questions regarding the educational and financial inequality in sport participation. First, we questioned whether educational and financial differences in the likelihood of continuous sport participation over the course of the COVID-pandemic exist. We conclude that the higher educated and people with no financial problems are more likely to report continuous sport participation in the 2019–2022 period compared to lower educated and those with financial problems. Second, we investigated the likelihood of bouncing back to sports in 2022 after the COVID-pandemic. Our results showed that higher educated were far more likely to bounce back than lower educated respondents. All in all, our main conclusion drawn from these results is that educational and financial inequality in sport participation further increased even after relaxation of the COVID-restrictions. We consider this to be an important conclusion, especially because a vast body of research demonstrates the beneficial impact of sport participation (Hoekman et al., 2022).

To the best of our knowledge, until now there have been no studies directed at the long-term impact of the COVID-pandemic for social inequality in sport participation. However, some previous studies are available that pay attention to changes in physical activity during and immediately after the first lockdown in 2020. Some of these show that many people were unable to return to previous physical activity levels during relaxed periods (Bu et al., 2021; McCarthy et al., 2021), while others contrastingly indicated that people did return to their pre-lockdown levels of physical activity (Janssen et al., 2020; Mata et al., 2021). These studies, however, were conducted at the start of the COVID-pandemic and provide no information on possible middle- or long-term impact of COVID. Contrastingly, our study highlights that COVID impacted social inequality in sport participation more than 2 years after its onset.

In line with previous research on the impact of crises for inequality (Collins & Haudenhuyse, 2015; Roberts, 2015), we found relatively strong negative consequences for lower educated individuals and people with financial problems. It seems likely that people belonging to these groups possess less resources to mitigate the impact of COVID measures on the short and longer term. This is in line with our expectations grounded in Bourdieu’s concepts of capital and habitus (Bourdieu, 1984; Kraaykamp, 2002). Individuals with a stronger sport habitus or sporting capital (Rowe, 2018), indeed seem more determined and better able to uphold, adapt or restart their sport behaviour despite all the barriers that the COVID-pandemic erects. As a result, we observed an increased inequality in sport participation for lower educated and those experiencing financial deprivation.

Certainly, our research yields implications for policymakers seeking to promote sport participation. We have shown that vulnerable groups are most affected by the COVID-pandemic. Although we conducted this study during and after the COVID-pandemic, we consider results generalisable to other crises (see Collins & Haudenhuyse, 2015; Roberts, 2015). Consequently, we encourage policymakers to express ongoing attention to specific target groups to reduce inequality in sport participation, especially in times of crises. After relaxation of the COVID-measures, the financial crisis and high energy prices likely put pressure on sports participation of vulnerable groups. It thus seems important to intensify sport promotion policies to ensure that social inequality in sport participation is not enlarged even further.

A limitation of our study is that we only focus on developments in whether people participate in sport or not. Future research might want to investigate whether results would vary looking at the frequency of sport participation or the type of sports people perform (individual sports, team sports). Moreover, we also recommend to focus future research on youngsters in their sports participation during COVID. With the LISS data at hand, we were not able to include people younger than 16 years old. Recent research illustrated a substantial dropout of sport club membership of the 4–17 years old during the COVID-pandemic (Hoekman et al., 2023). So, it would be informative to look at inequality in the development of the sport behaviour for youngsters.

To conclude, our study enhances the understanding of long-term impact of the COVID-pandemic on inequality in sport participation in the Netherlands and thus may inform policymakers to intensify sport promotion policies. Especially during future crises, ongoing attention to specific target groups is required to tackle social inequality in sport participation.
